# Direct Evidence for Infection of *Varroa destructor* Mites with the Bee-Pathogenic Deformed Wing Virus Variant B, but Not Variant A, via Fluorescence *In Situ* Hybridization Analysis

**DOI:** 10.1128/JVI.01786-20

**Published:** 2021-02-10

**Authors:** Sebastian Gisder, Elke Genersch

**Affiliations:** a Department of Molecular Microbiology and Bee Diseases, Institute for Bee Research, Hohen Neuendorf, Germany; b Institut für Mikrobiologie und Tierseuchen, Fachbereich Veterinärmedizin, Freie Universität Berlin, Berlin, Germany; University of Texas Southwestern Medical Center

**Keywords:** *Varroa destructor*, deformed wing virus, honey bee, host switch, quasispecies, viral virulence

## Abstract

Deformed wing virus (DWV) is a bee-pathogenic, single- and positive-stranded RNA virus that has been involved in severe honey bee colony losses worldwide. DWV, when transmitted horizontally or vertically from bee to bee, causes mainly covert infections not associated with any visible symptoms or damage. Overt infections occur after vectorial transmission of DWV to the developing bee pupae through the ectoparasitic mite Varroa destructor. Symptoms of overt infections are pupal death, bees emerging with deformed wings and shortened abdomens, or cognitive impairment due to brain infection. So far, three variants of DWV, DWV-A, DWV-B, and DWV-C, have been described. While it is widely accepted that *V. destructor* acts as vector of DWV, the question of whether the mite only functions as a mechanical vector or whether DWV can infect the mite, thus using it as a biological vector, is hotly debated because in the literature data can be found that support both hypotheses. In order to settle this scientific dispute, we analyzed putatively DWV-infected mites with a newly established protocol for fluorescence *in situ* hybridization of mites and demonstrated DWV-specific signals inside mite cells. We provide compelling and direct evidence that DWV-B infects the intestinal epithelium and the salivary glands of *V. destructor*. In contrast, no evidence for DWV-A infecting mite cells was found. Our data are key to understanding the pathobiology of DWV, the mite’s role as a biological DWV vector, and the quasispecies dynamics of this RNA virus when switching between insect and arachnid host species.

**IMPORTANCE** Deformed wing virus (DWV) is a bee-pathogenic, originally rather benign, single- and positive-stranded RNA virus. Only the vectorial transmission of this virus to honey bees by the ectoparasitic mite *Varroa destructor* leads to fatal or symptomatic infections of individuals, usually followed by collapse of the entire colony. Studies on whether the mite only acts as a mechanical virus vector or whether DWV can infect the mite and thus use it as a biological vector have led to disparate results. In our study using fluorescence *in situ* hybridization, we provide compelling and direct evidence that at least the DWV-B variant infects the gut epithelium and the salivary glands of *V. destructor*. Hence, the host range of DWV includes both bees (Insecta) and mites (Arachnida). Our data contribute to a better understanding of the triangular relationship between honey bees, *V. destructor*, and DWV and the evolution of virulence in this viral bee pathogen.

## Abstract (precis)

Deformed wing virus (DWV) is a bee-pathogenic, originally rather benign, single- and positive-stranded RNA virus. Only the vectorial transmission of this virus to honey bees by the ectoparasitic mite *Varroa destructor* leads to fatal or symptomatic infections of individuals, usually followed by collapse of the entire colony.

## INTRODUCTION

The Western honey bee (Apis mellifera) is a generalist, prolific, and versatile pollinator that is also quite easy to manage. Honey bees are therefore the most widely used commercial pollinator in agriculture and thus make a significant contribution to global crop production. Unfortunately, the health and well-being of honey bees around the world is threatened by a wide variety of pathogens and parasites. Viruses, especially those transmitted by the ectoparasitic mite Varroa destructor, play a major role in the worldwide reported losses of honey bee colonies. The virus, which is most often implicated in mite-related colony losses, is deformed wing virus (DWV) and is considered the most important reemerging pathogen of honey bees (1–5). No effective measures to fight this devastating virus in honey bee colonies are in place. Therefore, understanding the pathobiology of this virus is the only thing that might help to develop strategies to control it.

DWV is an RNA virus with a single-strand positive-sense genome of about 11 kb belonging to the family *Iflaviridae* in the order *Picornavirales* (6, 7). A hallmark of this and other families of RNA viruses is that their RNA-dependent RNA polymerase has limited template-copying fidelity and lacks proofreading and repair function (for recent reviews on viral quasispecies, see references 8–10 and references therein). The resulting error-prone replication process generates an indefinite number of viral mutants, which form dynamic and complex mutant clouds each one characterized by a master sequence. Therefore, DWV does not exist as a clearly circumscribed viral species with a definable consensus sequence but as viral quasispecies occupying a more or less defined sequence space. The mutant clouds moving within this sequence space are constantly subjected to genetic variation, competition, and selection. These processes give viral quasispecies enormous adaptive potential, resulting in its capacity to easily spread between different organs within a host or to switch between different host species.

DWV causes covert as well as overt infections in honey bees and can be transmitted horizontally, vertically, and vectorially. Covertly infected bees do not show any obvious symptoms and typically result from horizontal or vertical transmission of DWV between bees (11). Overt DWV infections are characterized by pupal death, emerging bees exhibiting malformed wings, and cognitive impairment in adult bees due to DWV infection of the brain (12–16). The occurrence of overt DWV infections is closely linked to the vectorial transmission of DWV to the developing honey bee pupa through the ectoparasitic mite *V. destructor* (17, 18). However, the majority of mite-vectored DWV infections result in covert infections, indicating that the vectorial transmission of DWV is necessary but not sufficient to cause overt infections (19, 20). Among the factors discussed to contribute to the development of overt infections are the mites’ ability to suppress the bee’s immune response (21–24) or differentially virulent DWV variants selected by and transmitted through infected mites acting as a biological vector (12, 14, 20, 25). While it is widely accepted that DWV exists as a quasispecies and that different DWV variants (DWV-A, DWV-B, DWV-C) are circulating (1, 3, 26, 27), it is still controversial whether these variants differ in their virulence for individual bees and bee colonies and/or in host preference.

The host range of the virus species DWV, originally thought to be bee-specific, and the host preference of the various DWV variants are also very controversial. There is ample evidence in the literature supporting the hypothesis that *V. destructor* is an alternate host for DWV and that differentially virulent, presumably mite- and bee-related DWV variants exist. The DWV variant, which is now known as DWV-B (3), was originally termed *Varroa destructor* virus-1 (VDV-1) because it was isolated as virus replicating in the mite (28). In the following years, numerous studies using different molecular, reverse transcription-PCR (RT-PCR)-based approaches detected the negative strand of the DWV genome in mites or mite tissues, indicating active DWV replication in the mite (12, 14, 20, 25, 29–31). Some of these studies suggested or even provided evidence that it is DWV-B rather than DWV-A that can infect the mite (12, 20, 29, 30). Furthermore, DWV-B was shown to be more virulent than DWV-A for both individual bees and bee colonies (12, 32, 33).

However, there are almost as many studies to suggest the opposite, namely, that DWV does not replicate in mites, i.e., that mites are not hosts of DWV (34–36), and that existing DWV variants are equally virulent (37) or that DWV-A is the more virulent variant (3, 38). The existing RT-PCR-based results on the replication of DWV in mites (12, 14, 20, 25, 28, 29) were negated, and it was suggested that they result from residual virus replication in ingested bee cells (3) or self-priming of positive-strand RNA during reverse transcription or random priming by tRNAs (39).

Whether or not DWV or certain variants of this virus can replicate in mites is crucial for understanding the pathogenesis of DWV infections and the relationship between *V. destructor* and the different virus variants. When RT-PCR-based molecular approaches are not convincing enough, direct and compelling evidence for DWV infection of mites is urgently needed. The aim of our study was therefore to provide this direct, nonmolecular evidence for DWV infection of mites. Our previous studies had shown that DWV does not replicate in all mites but only in part of the mite population infesting a colony (12, 14, 20, 25). Therefore, for our project on DWV replication in mites, we first identified a colony harboring a mite population with a high proportion of DWV-infected mites. We established a fluorescence *in situ* hybridization (FISH) analysis protocol for mites in order to be able to detect DWV directly inside mite cells as visual proof of infection. Using this protocol, we analyzed mites that were DWV infected according to prior RT-PCR analysis to see if we could, in fact, provide the missing direct evidence for DWV infection in mites and identify mite tissues targeted by DWV.

## RESULTS

### Identification of bee colonies suitable for sampling DWV-infected mites.

**FIG 1:**
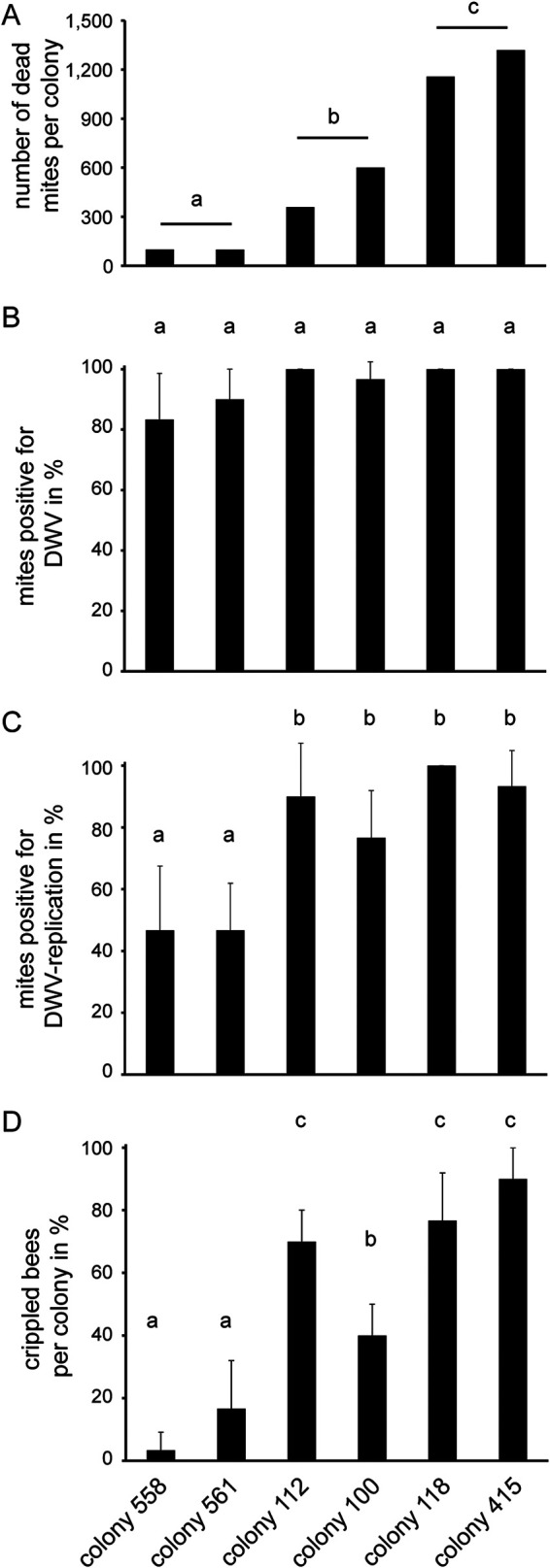
Mite status and proportion of crippled bees of the six colonies, no. 558, no. 561, no. 112, no. 100, no. 118, and no. 415. (A) Natural mite dead fall was determined over a time period of 7 days (21 to 28 August). (B) Proportion of DWV-positive mites on 28 August. From each colony, 30 mites were analyzed for the presence of DWV by using one-step RT-PCR. (C) Proportion of DWV-infected mites on 28 August. From each colony, 30 mites were analyzed for DWV infection by a two-step RT-PCR for detection of the DWV-negative strand. (D) Proportion of crippled bees per colony on 28 August. From each colony, 30 emerging worker bees were analyzed. Bars represent mean + standard deviation (SD), and different letters on top of bars represent statistically different groups.

In order to identify a honey bee colony that would be a suitable donor for DWV-infected *V. destructor* mites, we selected six mite- and virus-free colonies at the beginning of the bee season, which were managed throughout the season without any treatment against *V. destructor* to allow the undisturbed development of the mite populations and evolution of DWV quasispecies in these colonies. In late August, when the first crippled bees appeared in these colonies, we determined the mite infestation rate by counting the natural mite dead fall over 7 days (21 to 28 August). The six colonies split into three significantly different groups (analysis of variance [ANOVA], Fisher’s least significant difference [LSD], *P* < 0.05) exhibiting low (<100 mites), medium (330 to 600 mites), and high (>1,000 mites) infestation rates (Table 1; Fig. 1A). The proportion of DWV-positive mites (3 × 10 mites collected from the bottom board) did not differ significantly (ANOVA, Fisher’s LSD, *P* = 0.146) between the six colonies; 83.3% to 100% of the analyzed mites tested positive via RT-PCR for the DWV-positive strand (Table 1; Fig. 1B). However, there were significant differences (ANOVA, Fisher’s LSD, *P* = 0.005) between the colonies with respect to the proportion of DWV-infected mites (3 × 10 mites collected from the bottom board), i.e., which tested positive for the replicative negative strand of DWV via tagged strand-specific RT-PCR (20) (Table 1; Fig. 1C). In the two colonies with low infestation levels, only approximately 50% of the mites were DWV infected, while in the other four colonies, up to 100% of the tested mites were DWV infected. Three of these colonies also had a high proportion of crippled bees (70% to 90%; Table 1 and Fig. 1D), while one of these four colonies had a significantly lower (ANOVA, Fisher’s LSD, *P* ≤ 0.008) proportion of crippled bees (40%; Table 1 and Fig. 1D), which was still significantly higher (ANOVA, Fisher’s LSD, *P* ≤ 0.029) than the proportion of crippled bees in the two colonies which had a low mite infestation level and a low rate of infected mites (3% and 17%; Table 1 and Fig. 1D). Colony no. 118 with a proportion of 100% DWV-infected mites (Table 1) was the most suitable source colony for the analysis of DWV infection in mites, since it could be assumed that every mite in this colony is DWV infected.

**TABLE 1:**
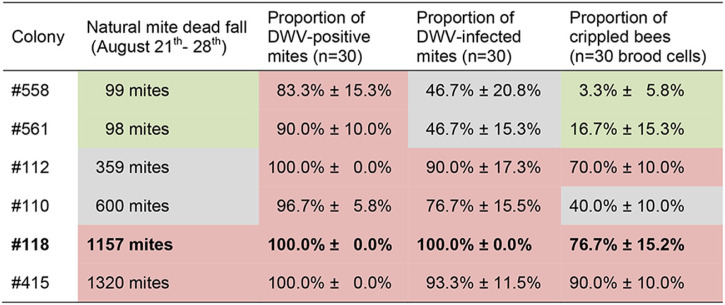
Natural mite dead fall, proportion of DWV-positive mites, proportion of DWV-infected mites, and proportion of crippled bees of the six analyzed colonies (558, 561, 112, 100, 118, and 415)^*a*^

a The colonies were managed without treatment against *V. destructor* during the honey bee season, and all samples were taken on 28 August.

### Identification of mite organs and tissues in cross sections.

**FIG 2:**
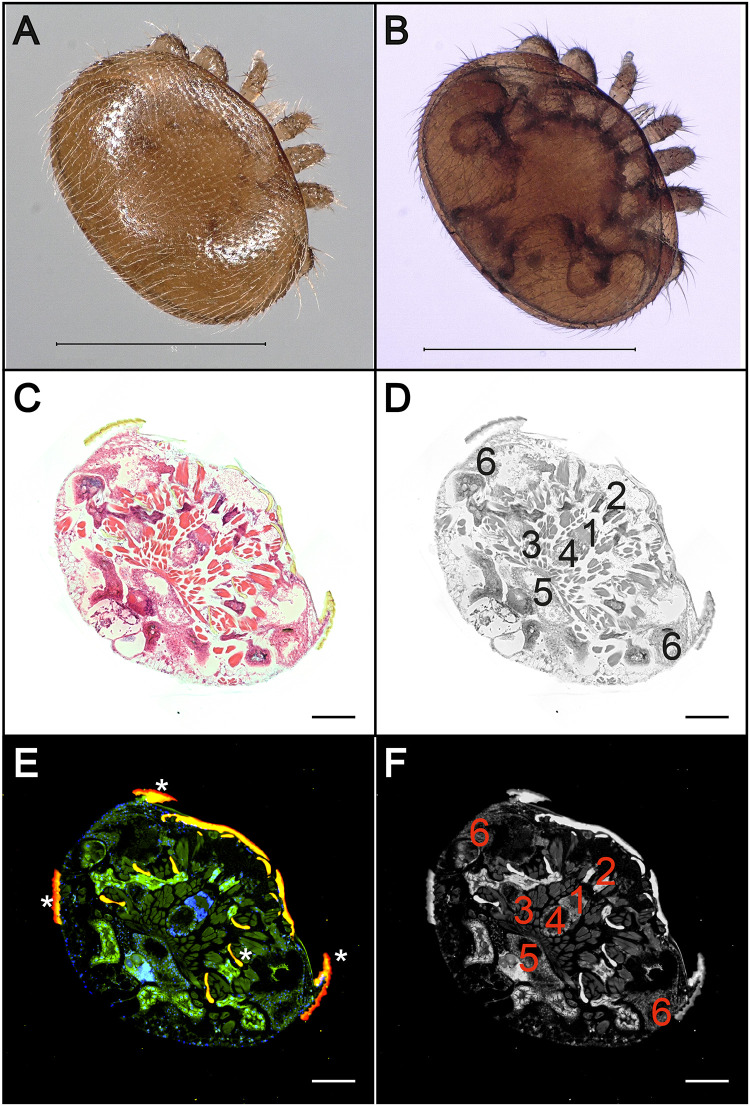
Anatomy and histology of adult female *V. destructor* mites. Dorsal (A) and ventral (B) pictures of an adult a female *V. destructor* mite. (C) H&E staining of a cross section of an adult female mite. (D) False-gray picture of the H&E-stained cross section to better mark the organs. (E) Fluorescence *in situ* hybridization (FISH) of a cross section of an adult female mite using an FITC-labeled Varroa-specific 18S rRNA probe (green fluorescence). Cell nuclei were stained with DAPI (blue signal). Autofluorescence is highlighted with asterisks. (F) False-gray picture of the FISH-analyzed cross section to better mark the organs. Both methods allow the localization of the brain (1), salivary glands (2), individual muscle bundles (3), ventriculus (4), lower gastric cecum (5), and upper gastric cecum (6) in the sections. Large images of entire mites in panels C through F were assembled with 3 by 3 single images at ×100 magnification. Representative pictures are shown. Bars represent 1 mm (A, B) and 200 µm (C to F).

Although there are innumerable publications on the biology and parasitic life style of *V. destructor*, the anatomy and histology of this mite are only described in rudimentary form (40, 41). Since the aim of this study is not only to provide direct evidence of DWV infection in *V. destructor* but also to identify the infected organs, we first wanted to get an overview of the mite anatomy with the help of hematoxylin-eosin (H&E) stains and fluorescence *in situ* hybridization (FISH) analyses using *V. destructor*-specific probes. In H&E-stained cross sections of adult mites, brain, salivary glands, individual muscle bundles, ventriculus, lower gastric cecum, and upper gastric cecum were easily identified (Fig. 2A and B). These organs and structures were also detectable in mite sections after FISH analysis performed with fluorescence-labeled probes hybridizing to *V. destructor*-specific regions of the mite’s 18S rRNA (Fig. 1C and D). Therefore, in the cross sections of the mites, all organs were recognizable which, according to the DWV tissue tropism in bees (12, 13, 42), could possibly also be infected in the mite: intestine, brain, and salivary glands.

### Detection of DWV infection in the intestine and salivary glands of *V. destructor*.

**FIG 3:**
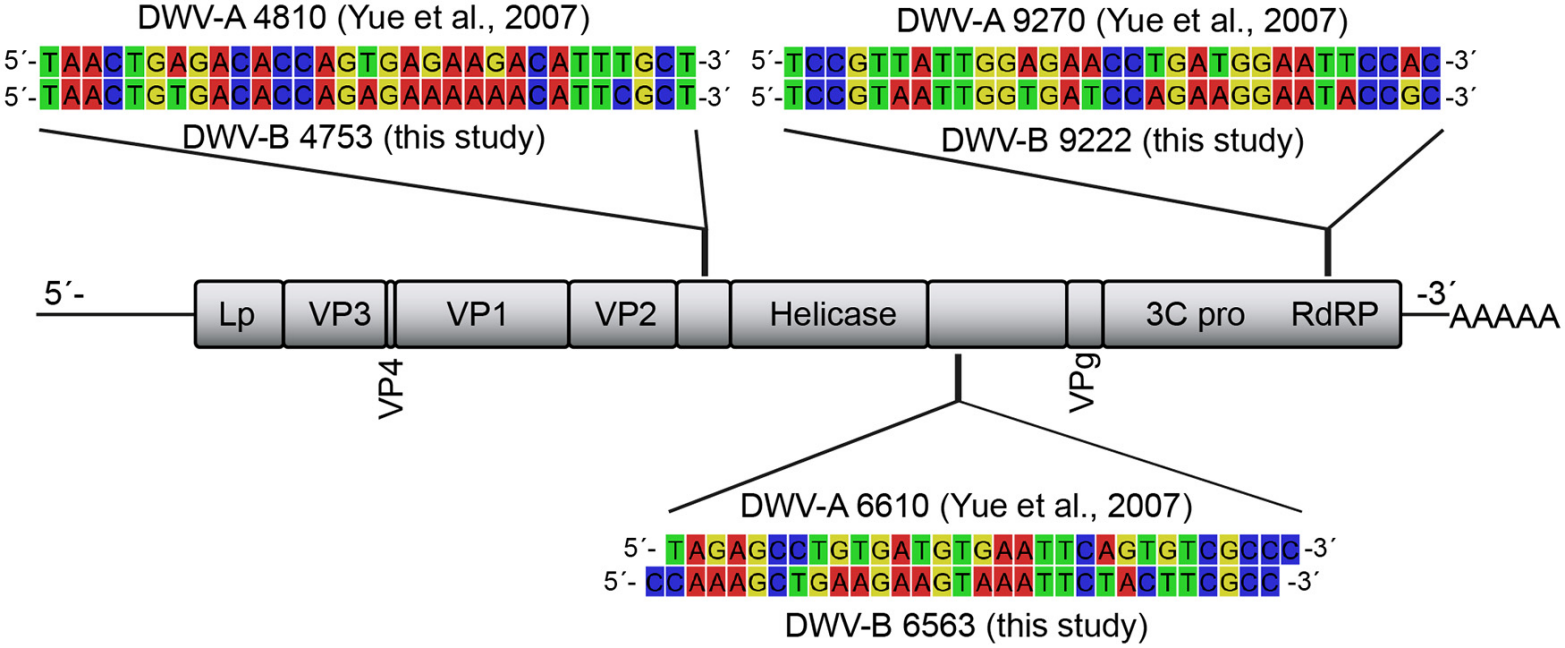
Alignment and localization of the DWV-A- and DWV-B-specific oligonucleotide probes that were used in this study. The oligonucleotides were designed according to the deposited reference genomes of DWV-A (GenBank accession no. NC_004830.2) and DWV-B (GenBank accession no. NC_006494). According to the genome organization of DWV, the oligonucleotides DWV-A 4810, DWV-A 6610, DWV-B 4753, and DWV-B6563 hybridize to noncoding regions, and the oligonucleotides DWV-A 9270 and DWV-B 9222 hybridize to the RNA-dependent RNA-polymerase (RdRP) gene.

**FIG 4:**
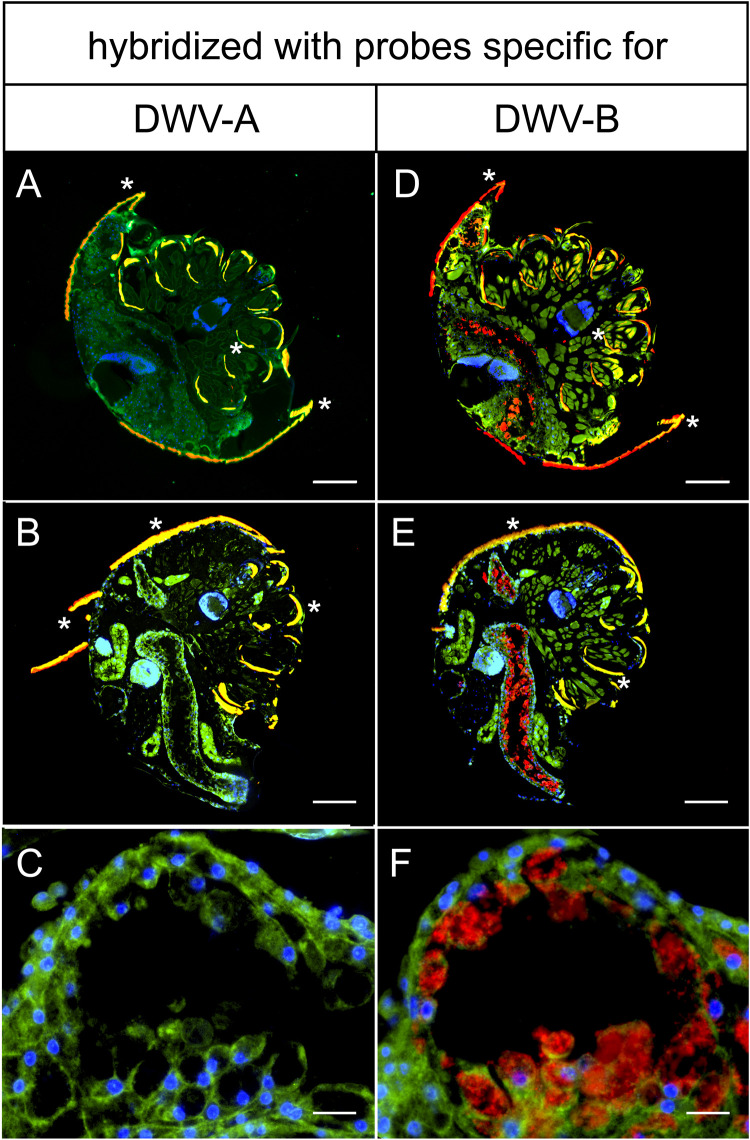
FISH-analysis of cross sections of DWV-infected female *V. destructor* mites. Mite sections were analyzed via FISH using 5′-TexasRed-labeled DWV-A-positive-strand-specific (A to C) or DWV-B-positive-strand-specific (D, E) oligonucleotides (both red fluorescence) and a *V. destructor*-specific 5′-FITC-labeled 18S rRNA-targeted (green fluorescence) oligonucleotide probe. Cell nuclei were stained with DAPI (blue signal). Mite cells appear in green with blue nuclei, and DWV-specific signals appear as red signals. Representative sections of mites are shown. Panels A and D as well as panels B and E are consecutive sections. Panels C and F are enlarged from Panels B and E, respectively. Autofluorescence of mite dorsal shields and legs is indicated by asterisks. Large images (A to D) of entire mites were assembled with 3 by 3 single images at ×100 magnification. Representative pictures are shown. Bars in panels A, B, D, and E represent 200 µm, and bars in panels C and F represent 20 µm.

Having identified a suitable source colony for DWV-infected mites and established a FISH protocol for analyzing mite sections, we started to provide direct evidence for DWV infection of mites. To this end, we had to confirm the presence of DWV inside of mite cells and to rule out mere acquisition of DWV in the mite’s gut lumen. To demonstrate DWV-infected mite cells, we first carried out FISH analyses with DWV-specific probes (Fig. 3), which had successfully been used in 2007 to detect DWV infections in the terminal part of a queen’s ovaries (11). At that time, these probes were designed using the DWV sequence published by Lanzi and coworkers (6), which we now classify as a DWV-A sequence (1, 3). Using these DWV-A-specific probes, no signals (red fluorescence) inside of mite cells that would indicate an infection of these cells were obtained in any of the mites examined (Fig. 4A to C). Since we hypothesized that the DWV variant replicating in the mites is DWV-B (12, 28), we next analyzed consecutive cross sections by using probes that bind in exactly the same DWV genome regions as the DWV-A-specific probes but are DWV-B specific (Fig. 3). This time, clear red fluorescence signals in the gastric ceca and the salivary glands were evident (Fig. 4D to F). While the signals in the gastric ceca (Fig. 4D and E) could be interpreted as coming from the gut lumen, the higher magnification of this area clearly showed that they were inside the epithelial cells lining the ceca (Fig. 4F).

**FIG 5:**
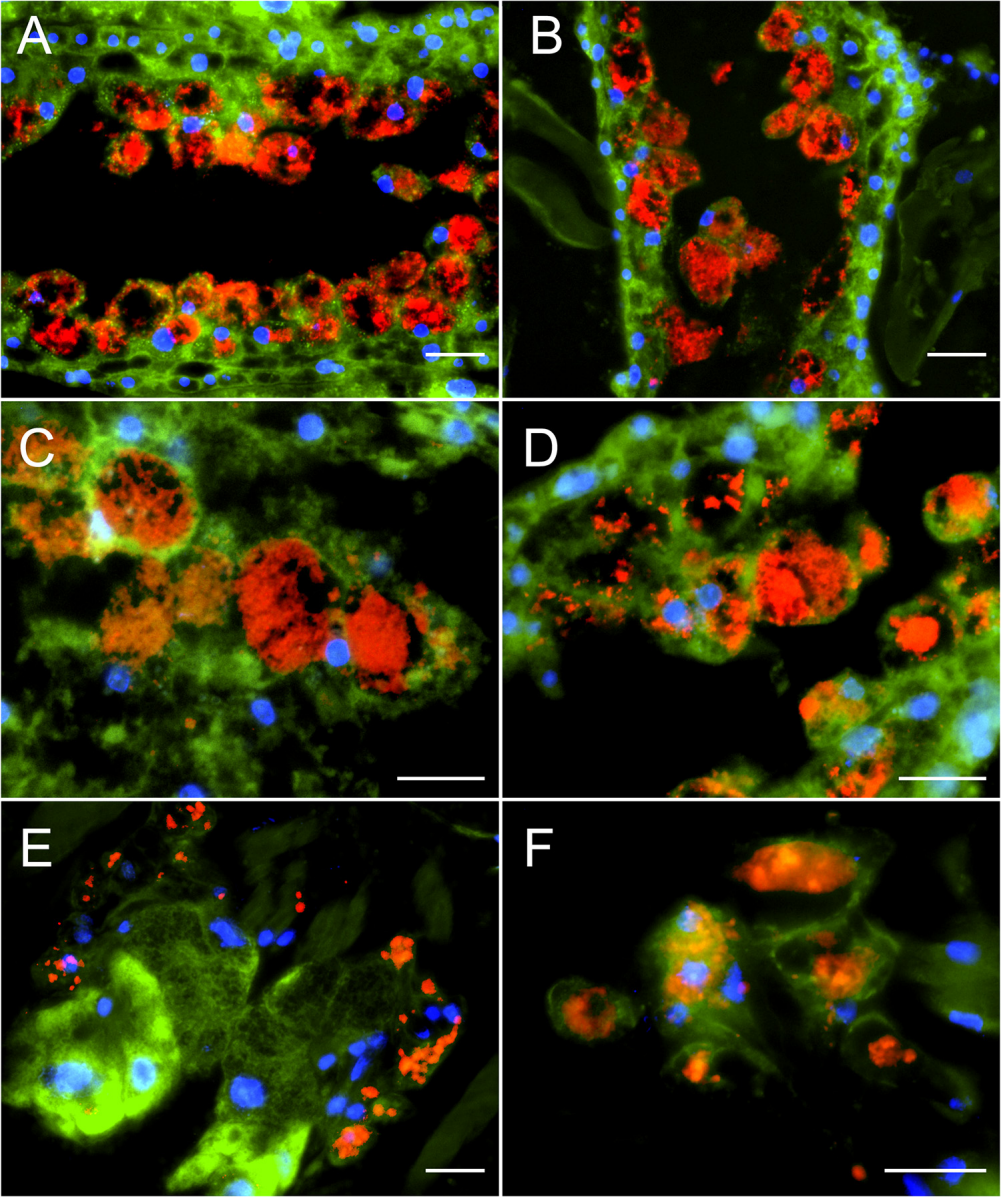
FISH analysis of DWV infecting the cecum and salivary glands of *V. destructor* mites. Representative results of DWV-B-specific FISH analysis of gastric ceca (A to D) and salivary glands (E and F) of DWV-B-infected mites are shown. Mite sections were analyzed via FISH using TexasRed-labeled, DWV-B-positive-strand-specific oligonucleotides (red fluorescence) and a *V. destructor*-specific 18S rRNA-targeted oligonucleotide probe (green fluorescence). Eukaryotic nuclei were stained with DAPI (blue fluorescence). Fluorescence signals were visualized by fluorescence microscopy at ×200 magnification (A, B, and E) and at ×400 magnification (C, D, and F). Representative pictures are shown. Scale bars represent 20 µm.

The localization of the DWV-B-specific fluorescence signals inside mite cells was further substantiated by detailed images from the ceca (Fig. 5A to D) and the salivary glands (Fig. 5E and F) of other examined mites. DWV-B-specific signals were clearly localized inside the epithelial cells lining the ceca and inside the salivary gland cells and were, therefore, direct evidence of an infection of mite cells with DWV-B. The infection of epithelial cells in the mites’ digestive tracts matches the assumed infection of the mites by virus particles ingested orally from infected pupae or bees. The infection of the salivary glands suggests that the retransmission of DWV to pupae or bees is also likely to occur orally via saliva containing infectious DWV-B originating from infected mite salivary glands. We found no evidence of DWV infection in the mites’ brains, although a recent publication assumed DWV-B infection of the mite synganglion (29).

## DISCUSSION

Numerous publications using RT-PCR-based approaches reproducibly demonstrated negative-strand DWV in mites, indicating active DWV replication in mites (12, 14, 20, 25, 29, 30, 43). However, the reliability of this molecular data was repeatedly questioned, and it was requested that instead methods such as FISH analyses must be used to separate true DWV infections of mites from DWV contaminations on the body surface or mere virus particle accumulation in the gut content (39). Here, we provide the requested proof. Our results obtained via FISH analyses clearly demonstrate DWV-B infection in mites and identify gut epithelium and salivary glands as target tissues for DWV-B infection. The gut epithelium as target tissue for DWV-B in mites is consistent with oral infection of the mite while it feeds on DWV-infected pupae and adult bees. Infected salivary gland tissue in mites indicates that DWV-B is transmitted back orally to parasitized bees, thus contributing to the spread of DWV-B in the bee population.

The following question remains: why did we find DWV-infected mites while others found no evidence of DWV infection in mites? The simplest explanations are that not every mite is DWV infected (12, 14, 20, 25) and that not all DWV variants infect mites (20, 29). In most colonies, the infesting mite populations are quite heterogeneous and consist of both DWV-negative and DWV-positive mites, and again among the DWV-positive mites, there are both infected and noninfected mites (14, 20, 25; this study). Therefore, depending on the proportion of infected mites in a given colony, the probability to sample an infected mite varies and can be quite low. However, it is possible to enhance the chances to sample infected mites for further analyses by using established strand-specific RT-PCR protocols for prescreening of the mite populations for presence of DWV replication in mites prior to applying other methods like immunohistochemistry (34), proteomics (35), or FISH analysis (this study). Alternatively, since infected mites acting as biological DWV vector are strongly associated with overt infections in the developing, parasitized bee (20), infected mites can be sampled upon emergence of crippled bees from their brood cells (25). In the two studies most often cited as proof against DWV replication in *V. destructor* (34, 35), the mite populations were neither prescreened nor were the mites sampled from emerging crippled bees; therefore, chances are high that the authors just missed the infected mites. Another publication (29) is often wrongly cited as having “failed to detect any negative-strand DWV in any of the mite tissues assayed” (39). In fact, Campbell and coworkers reported that by applying DWV variant- and strand-specific reverse transcriptase quantitative PCR (RT-qPCR), they detected the presence of negative-strand RNA from VDV-1 (i.e., DWV-B) in all tissues assayed but failed to detect negative-strand DWV (i.e., DWV-A) (29). Unfortunately, in their publication, they used the terms DWV and VDV-1 instead of DWV-A and DWV-B, respectively. This use of the already at that time obsolete nomenclature for the DWV variants leads to confusion and to the fact that the results, that only DWV-B but not DWV-A was found replicating in mites, are not fully recognized.

Our results obtained with FISH analysis now further substantiate that the described variants of DWV are differentially able to infect mites. We did not obtain any DWV-specific signal inside mite cells when using probes specific for DWV-A, indicating that DWV-A does not infect mites. This result is consistent with a recent study proposing that DWV-A is transmitted through *V. destructor* in a nonpropagative manner (36). In contrast, when applying DWV-B-specific probes, strong signals were visible inside the epithelial cells lining the ceca and in the salivary gland cells. These results provide direct evidence that DWV-B can infect these mite tissues and suggest that being able to replicate in mites and thus infect mites is not a common characteristic of all members of the quasispecies DWV but rather restricted to DWV-B. Therefore, analyzing noninfected mites or analyzing infected mites with primers or probes for DWV-A will give negative results for DWV replication in mites (34, 35), whereas analyzing infected mites with primers/probes (also) detecting DWV-B will allow the detection of DWV infection in mites (12, 14, 20, 25, 29).

When we first published about DWV replication in mites (20) and about the strong association between DWV replication in mites and the occurrence of crippled wings in bees developing from pupae infested by DWV-infected mites (20, 25), we already discussed the possible existence of two different subpopulations of DWV differing in their capacity to replicate in mites and presumably also differing in virulence. We also already then hypothesized that VDV-1 (28) (now called DWV-B) is the most likely candidate for this mite-infecting DWV subpopulation (20). Our results presented here, together with the results of Campbell and coworkers (29), ultimately provide direct evidence for the following 15-year-old hypothesis: VDV-1, originally isolated as picorna-like, mite-infecting virus (28), later assigned as a member of the DWV quasispecies (19) and renamed DWV-B (3), is the proposed DWV subpopulation (20) or variant that is adapted to replicating in the mite and may be associated with increased virulence in pupae and bees (12, 32, 33).

Based on our previous results (12, 14, 20, 25) and the FISH results presented in this study, which substantiate the role of *V. destructor* as alternate host for DWV-B but not for DWV-A, we propose that these two (and presumably other) variants evolved when DWV, an insect virus, was confronted with a new potential host, the mite *V. destructor*, and expanded its host range to include this arachnid. Every switch from the insect to the arachnid host and back again presumably represents an evolutionary bottleneck for the infecting DWV mutant clouds resulting in the selection of DWV-B- or DWV-A-like founder viruses adapted to replication in mites or bees, respectively. Error-prone replication of these founder viruses in the mite or bee host will again generate a mutant cloud that contains mutants capable of switching the host again. In order to fully understand how DWV manages to move between the honey bee and the mite host, we need to identify the relevant DWV genome sequence and protein signatures, which determine host specificity and tissue tropism. We propose that the presence of these molecular signatures regardless of the overall genomic context (DWV-A- or DWV-B-like) determines host specificity and virulence and that detecting these signatures will allow a much better assessment of DWV host specificity and virulence than the overly general classification into the known DWV variants.

## MATERIALS AND METHODS

### Bee and mite material.

All bee and mite material used in this study originated from *A. mellifera* colonies of the Institute for Bee Research in Hohen Neuendorf, Germany. Mid-March 2019, at the beginning of the honey bee season, 16 honey bee colonies that did not show any signs of overt DWV infections (crippled bees) were screened for DWV, sacbrood virus (SBV), chronic bee paralysis virus (CBPV), and Kashmir bee virus (KBV) infections by molecular diagnostics. To this end, larvae, pupae, and adult worker bees (10 individuals each) were sampled from each colony, and virus diagnostic was performed as previously described (12, 13). Briefly, RNA was extracted from larvae or heads of decapitated pupae and worker bees by using the RNeasy minikit from Qiagen (Hilden, Germany) according to the manufacturer’s protocol. Qualitative one-step RT-PCR analysis was performed as previously described (13). Six virus-free colonies (no. 558, no. 561, no. 112, no. 100, no. 118, and no. 415) were identified, which were also mite-free at this time point. These colonies were then managed without *V. destructor* treatment for the entire study period to allow undisturbed development of the mite populations and DWV quasispecies. Mites serving as negative control for establishing the FISH protocol were collected at the beginning of the bee season from a virus-free honey bee colony that was infested by a mite population that tested negative for DWV via RT-PCR at the time point of sampling.

### Analysis of the colony status.

The *V. destructor* mite infestation status of the six colonies (Fig. 1A) was determined at the end of August (21 to 28 August) when the first crippled bees were observed in the colonies. To this end, debris was examined for the presence of natural mite dead fall as previously described (44). Briefly, the hives were equipped with clean bottom boards for 1 week, and natural mite dead fall on the bottom board was counted after removing the bottom boards.

The DWV status of the mite populations in the six colonies was determined on 28 August, when the bottom boards were removed, by collecting mites from each bottom board. From each colony, 30 dead mites, located at the upper left (*n* = 10), the middle (*n* = 10), and the lower right (*n* = 10) area of the boards, were sampled individually in 1.5-ml reaction tubes (Eppendorf), and the proportion of DWV-positive and DWV-infected mites was analyzed. To this end, extraction of total RNA from *V. destructor* mites as well as qualitative one-step RT-PCR analysis for the detection of the DWV-positive strand (mites positive for DWV) (Fig. 1B) and qualitative, tagged two-step RT-PCR analysis for the detection of the DWV-negative strand (mites positive for DWV infection) (Fig. 1C) were performed essentially as previously described (20, 25, 45).

The proportion of crippled bees (Fig. 1D) was also determined on 28 August when the bottom boards were removed. To this end, emerging bees or bees about to emerge were collected from one brood frame per colony as recently described (12, 25). Groups of 10 bees per colony were sampled with a defined lag period of 30 min between the three samplings. Briefly, as soon as an emerging bee started to open the brood cell, the wax cap was removed with forceps and the honey bee was carefully removed from the cell. Bees showing symptoms of overt DWV infection like wing deformities were classified as crippled bee.

### Embedding and sectioning of *V. destructor* mites.

Colony no. 118 harboring a mite population with a high proportion of DWV-infected mites (according to RT-PCR analysis), was used as the source colony for mites to be analyzed with fluorescence *in situ* hybridization (FISH). Several samples of 50 to 100 adult worker bees were collected in 50-ml Falcon tubes (VWR, Darmstadt, Germany), and bees were immobilized on ice for 10 min. Phoretic mites were removed from the bees with forceps and individually transferred into 1.5-ml reaction tubes (Eppendorf) for fixation with 1.0 ml of 4% Roti Histofix (Carl Roth, Karlsruhe, Germany) for 2 h at room temperature (RT). Subsequently, the mites were washed with 1.0 ml of 6.8% sucrose in phosphate-buffered saline (PBS) (pH 7.0) overnight at RT, followed by a dehydration step with 100% acetone for 8 h at RT with gentle agitation on a shaker. The mites were preinfiltrated with 1.0 ml of 50% acetone/50% Technovit 7100 (Kulzer, Wehrheim, Germany) overnight at RT and subsequently infiltrated with 1.0 ml Technovit 7100 infiltration solution supplemented with 1% hardener I (wt/vol) for 24 h at RT with gentle agitation. Next, the mites were incubated in 500 µl of 14% sodium hypochloride (VWR, Darmstadt, Germany) for 60 min with gentle agitation followed by three washing steps with Technovit 7100 infiltration solution. For embedding, infiltrated mites were carefully placed in precooled (−20°C) histoforms. The histoform wells were filled with 1.0 ml Technovit 7100 resin (infiltration solution supplemented with 6.66% [vol/vol] hardener II) and were incubated overnight at 4°C to allow gentle polymerization. Next, the embedded tissue was blocked with Technovit 3040 resin according to the manufacturer’s protocol. Histological semithin sections (8 to 10 µm) were prepared by using a rotary microtome (Thermo Fisher Scientific, Walldorf, Germany) and a tungsten carbide knife with a D profile (Leica, Wetzlar, Germany). All native histological sections were fixed on glass slides with tap water and stored at 4°C until further processing.

### Hematoxylin-eosin staining of mite tissue sections.

Native mite sections were stained with the modified hematoxylin-eosin (H&E) fast staining kit (Carl Roth, Karlsruhe, Germany) according to the manufacturer’s protocol. Briefly, sections were stained with H&E solution 1 for 6 min and rinsed with tap water for 10 s. Subsequently, the tissue was covered with 0.1% hydrochloric acid, and after incubation for 10 s, the slide was rinsed with tap water for 6 min. H&E solution 2 was added to the slides and was removed after 30 s by rinsing with tap water for another 30 s. Stained mite sections were air dried and covered with a cover slip by using Entellan (VWR, Darmstadt, Germany).

### Fluorescence *in situ* hybridization analysis of mite tissue sections.

FISH analysis was performed by using 5′-TexasRed (sulforhodamine 101 acid chloride; TR) oligonucleotides (Eurofins Genomics, Ebersberg, Germany) as probes for the specific detection of DWV-A or DWV-B RNA (Table 2; Fig. 3). Three oligonucleotide probes hybridizing to three different regions to the DWV-A (DWV-A 4810, DWV-A 6610, and DWV-A 9270) or DWV-B (DWV-B 4753, DWV-B 6563, and DWV-B 9222) genomes were used (positions according to GenBank accession no. NC_004830.2 and NC_006494.1). All virus-specific oligonucleotides represent antisense probes, which hybridize to the positive strand of the DWV-A or DWV-B genomes. A 5′-TexasRed-labeled nonsense oligonucleotide (nonsense) (Table 2) was used as control to exclude false-positive results by unspecific hybridization reactions.

**TABLE 2:** Oligonucleotide probes used in this study for specific detection of DWV-A or DWV-B genomes or *Varroa destructor* 18S rRNA

Oligonucleotide name	Accession no. ofreference sequence	Sequence 5′–3′	Position	5′-fluorescence dye	Reference or source
DWV-A 4810	NC_004830.2	TAACTGAGACACCAGTGAGAAGACATTTGCT	4,780–4,810	TexasRed	11
DWV-A 6610		TAGAGCCTGTGATGTGAATTCAGTGTCGCCC	6,580–6,610	TexasRed	11
DWV-A 9270		TCCGTTATTGGAGAACCTGATGGAATTCCAC	9,240–9,270	TexasRed	11
DWV-B 4753	NC_006494.1	TAACTGTGACACCAGAGAAAAAACATTCGCT	4,753–4,783	TexasRed	This study
DWV-B 6563		CCAAAGCTGAAGAAGTAAATTCTACTTCGCC	6,563–6,593	TexasRed	This study
DWV-B 9222		TCCGTAATTGGTGATCCAGAAGGAATACCGC	9,222–9,252	TexasRed	This study
Nonsense		GCGTAGTGCAAGCTGATCCGCTAGTGACTG		TexasRed	11
VdP1	FJ911866.1	CGGCAAAAACAGTTACCTATATTTGC	561–586	FITC	This study
VdP2		GTAAGCCAAGGCAAGTTTTCA	508–528	FITC	This study

To visualize the cytoplasm of the mite cells, 5′-FITC (fluorescein isothiocyanate)-labeled, *V. destructor*-specific oligonucleotides were used. The oligonucleotides Vd-P1 and Vd-P2 (Table 2) were designed according to the V4 region of the *V. destructor* 18S rRNA gene partial sequence (GenBank accession no. FJ911866.1).

For hybridization, mite sections were washed three times with 1× PBS (phosphate-buffered saline) in diethyl pyrocarbonate (DEPC)-treated water. To improve permeability of the mite sections, incubation with proteinase K (1 µg/ml) in 1× PBS for 10 min at 37°C was performed prior to hybridization. FISH was performed in Corning hybridization chambers (Merck, Darmstadt, Germany) with LifterSlips (Thermo Fisher Scientific, Schwerte, Germany) at 45°C for 24 h using 1 µg of each probe diluted in 50 μl of hybridization buffer (20% [vol/vol] deionized formamide, 0.9 M NaCl, 20 mM Tris‐HCl, pH 7.9, 0.01% [wt/vol] SDS) for each section. Subsequently, sections were washed six times with 1× PBS. Nonspecific autofluorescence of mite tissue was reduced by applying the Vector TrueView autofluorescence quencher kit from Vector laboratories (Vector Laboratories, Burlingame, CA, USA) according to the manufacturer’s protocol. Finally, sections were mounted with the Vectashield Vibrance antifade mounting medium with 4′,6-diamidino-2-phenylindole (DAPI). Bright-field and fluorescence microscopy was performed using an Eclipse Ti fluorescence microscope (Nikon, Düsseldorf, Germany) with a standard set of fluorescence filters.

**FIG 6:**
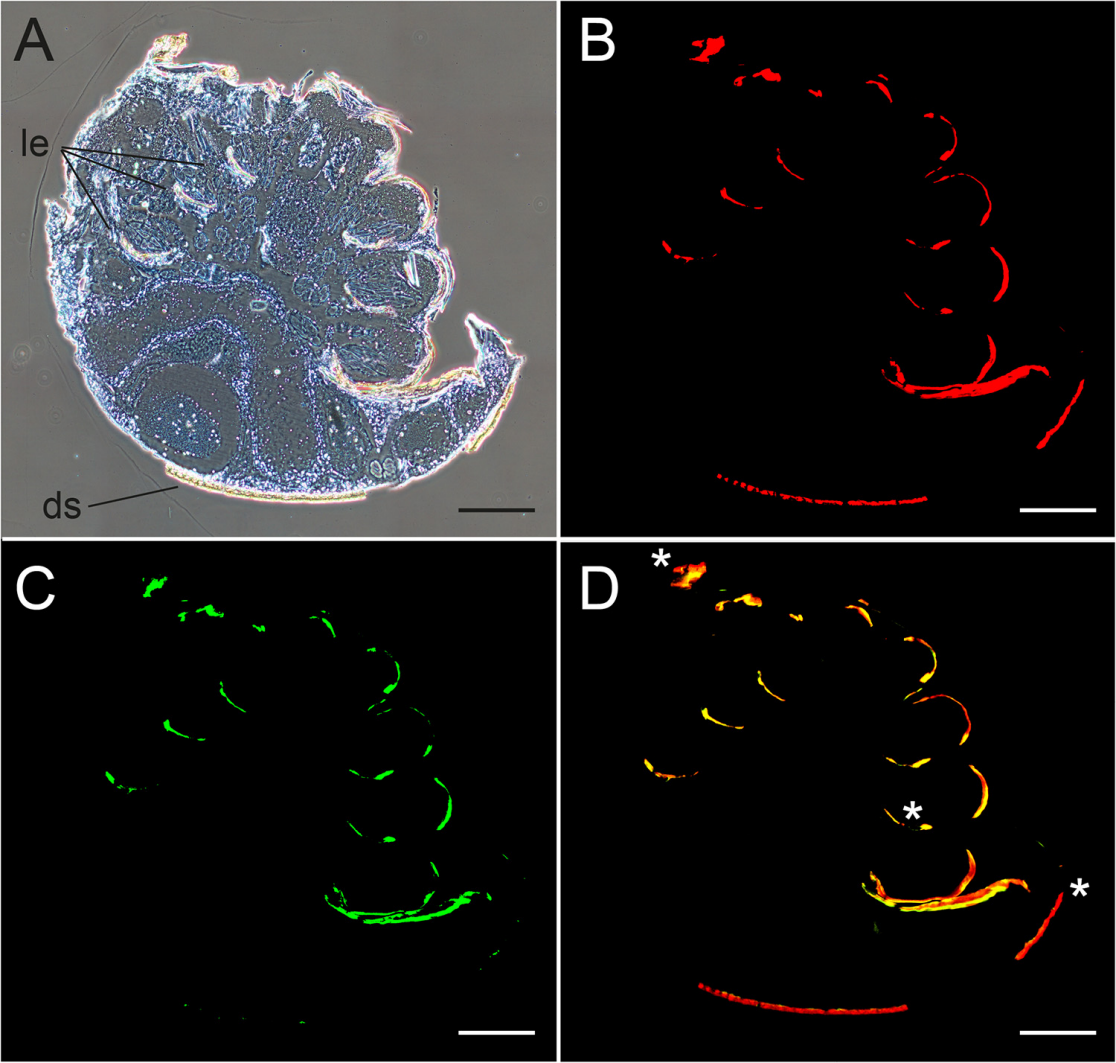
Analysis of cross sections of female *V. destructor* mites to demonstrate the autofluorescence of the cuticula. Mite sections were incubated only with hybridization buffers not containing any labeled oligonucleotides. (A) Bright-field microscopy with phase contrast visualizes the cuticula of legs (le) and dorsal shield (ds). (B) Fluorescence microscopy at 615 nm shows red autofluorescence of the cuticular structures of the mite’s dorsal shield and legs. (C) Fluorescence microscopy at 520 nm shows green autofluorescence of the cuticular structures of the mite’s dorsal shield and legs. (D) Merged fluorescence channels (B and C) resulting in an orange signal (*) by the overlay of red and green signals for autofluorescence of the dorsal shield and legs. To obtain images of entire mites, 3 by 3 single images at ×100 magnification were assembled. Representative pictures are shown. Bars represent 200 µm.

Bright-field microscopy of tissue sections showed that remains of the mites’ legs and dorsal shields still adhered to the semithin sections (Fig. 6A). As expected (46), these chitin-rich cuticular structures showed red autofluorescence at 615-nm (Fig. 6B) and green autofluorescence at 520-nm emission wavelengths (Fig. 6C). The overlay of both fluorescence channels resulted in an orange signal (Fig. 6D). Since the cuticular structures of the legs or dorsal shields could not be removed entirely from the mite sections analyzed for DWV infection via FISH, autofluorescence signals were indicated by asterisks in all further figures if present. The presence of the chitin autofluorescence, however, did not hamper the interpretation of the FISH analysis since the specific signals for DWV were inside the mite cells and not associated with cuticular structures.

**FIG 7:**
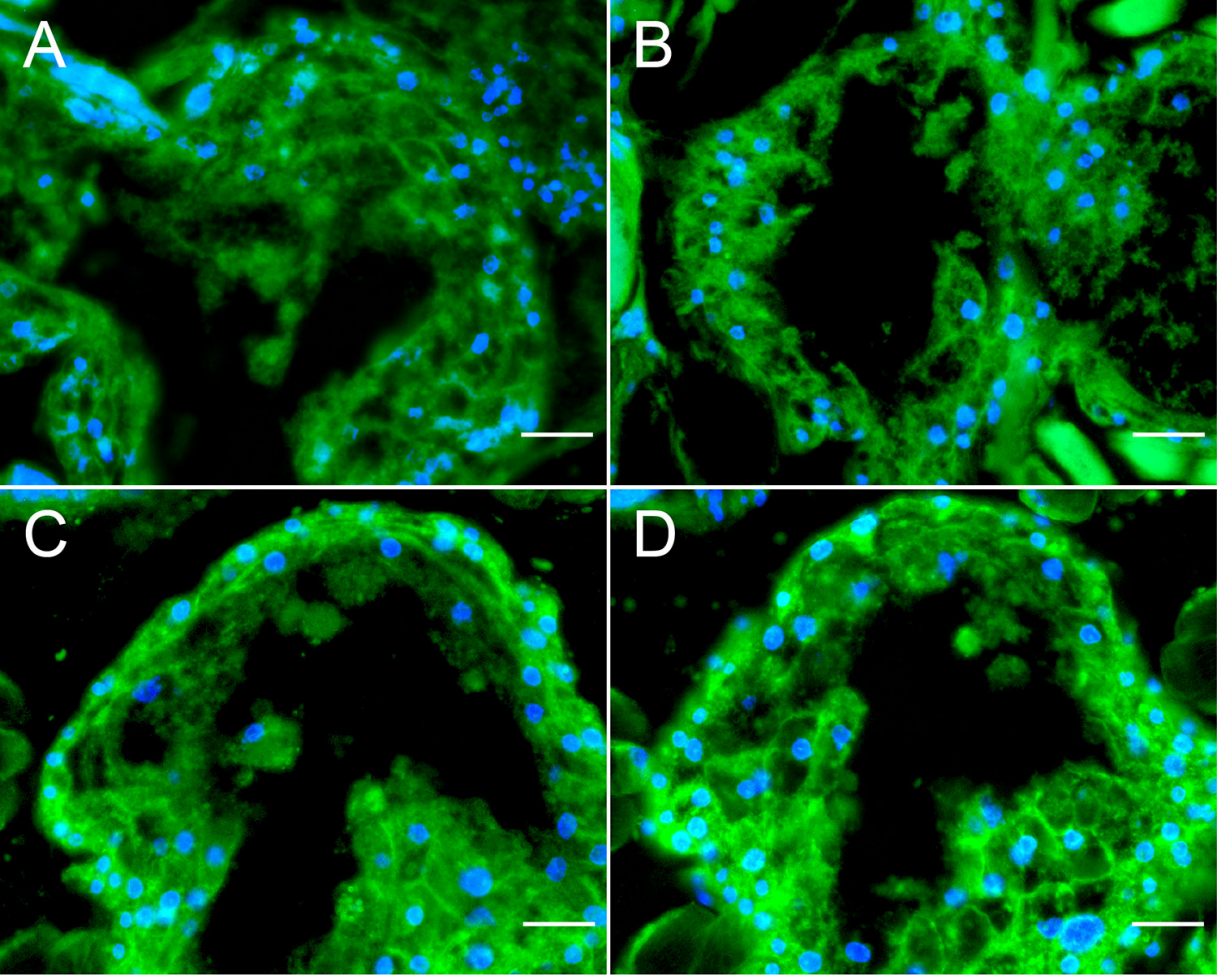
Negative controls for fluorescence *in situ* hybridization (FISH) analysis. Cross sections of female noninfected *V. destructor* mites were analyzed via FISH in the absence of any DWV-specific oligonucleotide (A) as well as by applying a 5′-TexasRed-labeled nonsense oligonucleotide probe (B), 5′-TexasRed-labeled DWV-A-positive-strand-specific oligonucleotides (C), or 5′-TexasRed-labeled DWV-B-positive-strand-specific oligonucleotides (D) (all three with red fluorescence). Mite tissue was visualized by applying 5′-FITC-labeled *V. destructor*-specific 18S rRNA-targeted oligonucleotide probes (green fluorescence). Eukaryotic nuclei were stained with DAPI (blue fluorescence). Fluorescence signals were visualized at ×200 magnification. Representative pictures of mite ventriculi are shown. Scale bars represent 20 µm.

The specificity of the used oligonucleotides was verified by FISH analysis of mites derived from a colony harboring a mite population that tested negative for DWV via RT-PCR (negative controls). FISH was performed without any oligonucleotide specific to DWV-A or DWV-B (Fig. 7A) or by applying the 5′-TexasRed-labeled nonsense oligonucleotide (Fig. 7B) or the 5′-TexasRed-labeled DWV-A-positive-strand-specific oligonucleotides (Fig. 7C) or the 5′-TexasRed-labeled DWV-B-positive-strand-specific oligonucleotides (Fig. 7D). No red signal was detected for any 5′-TexasRed-labeled oligonucleotide in these negative controls.

### Data analysis.

Mite infestation rates, proportion of DWV-positive mites, proportion of DWV-infected mites, and proportion of crippled bees were analyzed with a one-way ANOVA and Fisher’s least significant difference (LSD) as *post hoc* test using XLSTAT (statistical and data analysis solution, version 2020.1.3) software.
